# Male and female preferences for nest characteristics under paternal care

**DOI:** 10.1002/ece3.5363

**Published:** 2019-06-17

**Authors:** Varpu Pärssinen, Nadine Kalb, Martin Vallon, Nils Anthes, Katja Heubel

**Affiliations:** ^1^ Department of Biosciences University of Helsinki Helsinki Finland; ^2^ Tvärminne Zoological Station University of Helsinki Hanko Finland; ^3^ Department of Biology Lund University Lund Sweden; ^4^ Institute for Evolution and Ecology University of Tübingen Tübingen Germany; ^5^ Department of Biology University of Tübingen Tübingen Germany

**Keywords:** extended phenotype, mate choice, multiple cues, nest quality, parental investment, sexual conflict

## Abstract

Nests play a critical role for offspring development across the animal kingdom. Nest quality may contribute to the builder's extended phenotype and serve as an ornament during mate choice. We examined male and female nest choice in the common goby (*Pomatoschistus microps*), a benthic fish with male‐only parental care where females deposit eggs in male‐built nests. Using prebuilt nest models, we independently manipulated two candidate nest quality traits: (a) nest entrance width with a role in oxygen ventilation, and (b) extent of sand cover with a role in camouflage. In simultaneous choice trials, male gobies exhibited no preference for any nest model type. This suggests that initial characteristics of a nesting substrate have minor importance for males, which usually remodel the nest. Females were given a choice between two males occupying either entrance‐ or cover‐manipulated nests. The same pair of males was then exposed to a second female but now with alternated nest types assigned. Most females were consistent in choosing the same, typically the heavier male of the two regardless of nest properties. However, the females that chose the same nest regardless of the male preferred low over high sand coverage and narrow over wide nest entrance. Our results indicate that females base their mating decision on a combination of male phenotype and nest traits. While we found no indication that females are attracted to highly decorated nests, our study is the first in fishes to disentangle a preference for narrow (and thus more protective) nest entrances independent of nest coverage.

## 1 INTRODUCTION

Nests provide shelter for the developing young against environmental stress and predation (Bolnick, Shim, & Brock, 2015; Li et al., 2018; Morrell, Hentley, Wickens, Wickens, & Rodgers, 2012). A well‐built nest can not only decrease offspring mortality, but also reduce the need for parental care. For example, lesser kestrels (*Falco naumanni*) prefer used nesting sites with organic material, as these nests potentially reduce needed incubation effort as well as raise the hatching success of eggs (Podofillini et al., 2018). These direct benefits alone often make it beneficial to choose mates with well‐built nests (Alatalo, Carlson, & Lundberg, 1988; Evans, 1997; Grubbauer & Hoi, 1996; Quader, 2006; Sargent & Gebler, 1980). Nevertheless, also indirect benefits may explain nest choice. Nest building is a costly behavior and hence might signal the nest builders quality (reviewed in Mainwaring, Hartley, Lambrechts, & Deeming, 2014).

Especially in cases where only a single parent (typically the male) is responsible for nest building, the nesting site can additionally provide quality information about its architect (reviewed in Moreno (2012); Barber (2013)), thus contributing to an extended phenotype (Dawkins, 1982; Schaedelin & Taborsky, 2009). In this context, nest attributes may provide honest signals of direct benefits, for example, through improved parental care (De Neve, Soler, Soler, & Perez‐Contreras, 2004), and indirect benefits, for example, through elevated immune function (Soler, Martin‐Vivaldi, Haussy, & Moller, 2007). In birds, nests often contribute to postmating sexual selection, for example, by stimulating males to invest more feeding effort when females had invested more into nest building (Jelinek, Pozgayova, Honza, & Prochazka, 2016).

Mating decisions based on nest quality should only benefit the female if nests either provide sufficiently large direct benefits to offspring survival, or reliably serve as a proxy for male quality (Kokko, 1998). Whether either of these requirements is fulfilled depends on the environmental context, since benefits from a well‐built nest can be higher when predators are present (Candolin & Voigt, 1998), as well as depend on the costs of parental care (Lehtonen, Wong, & Kvarnemo, 2016; Ortiz‐Ceballos, Perez‐Staples, & Perez‐Rodriguez, 2016; Peluc, Sillett, Rotenberry, & Ghalambor, 2008; Thomas, Bateman, Scantlebury, & Bennett, 2012; Wong, Tuomainen, & Candolin, 2012) or the degree of mate competition (Heubel, 2018; Jordan, Maguire, Hofmann, & Kohda, 2016). Moreover, the nest may be more reliable as an extended phenotype if maintenance costs are high (Jordan et al., 2016), but less so if there is a risk that other individuals take over the nest (Bisazza & Marconato, 1988). Given such variation in the relevant costs and benefits, female mate choice may optimally draw from multiple independent cues (Bro‐Jørgensen, 2010; Candolin, 2003). Indeed, females have been shown to rely on different cues when environmental conditions vary (Chaine & Lyon, 2008; de Jong, Amorim, Fonseca, & Heubel, 2018). Similarly, the relevance of (more indirect) nest cues versus (more direct) male phenotype cues has varied between studies (Head, Fox, & Barber, 2017; Heubel, 2018). Many earlier studies investigating the role of alternative cues in mate choice face the challenge to clearly isolate the preference for nest quality from preference for male phenotypes, and none has independently controlled multiple nest traits when studying female choice (but see Bose et al., 2018).

The current study takes advantage of an established study system with male‐only care. The common goby (*Pomatoschistus microps*) is a small benthic short‐lived fish that builds nests underneath mussel shells or other solid objects by excavating a sand cavity below and gathering sand on top of the shell (Nyman, 1953). Both males and females mate repeatedly during a single reproductive season (Miller, 1975). Nest takeovers by competitors and nest‐loss due to waves and movement of sand may occur (Magnhagen, 1992; Mück & Heubel, 2018). Depending on the size of the nest, males may simultaneously accommodate clutches of one to three females (Mück & Heubel, 2018). Males attract females to attach eggs on the ceiling of their nest and provide paternal care for the eggs until hatching. Paternal care includes water ventilation by fanning with pectoral fins, as well as actively protecting the clutch from predators (Jones & Reynolds, 1999b), and removal of dead or infected eggs within the brood (Vallon, Anthes, & Heubel, 2016). Females have a preference for males with larger nests, but unlike in many other species with male parental care (Forsgren, Kvarnemo, & Lindström, 1996; Kraak & Groothuis, 1994; Requena & Machado, 2015), females do not always prefer nests that already contain eggs (Heubel, 2018, but see Reynolds & Jones, 1999). Offspring survival also varies with nest characteristics: A well‐constructed nest in terms of concealing sand cover and narrow entrance is less susceptible to predation from both the sand level (Jones & Reynolds, 1999b) and aerial predation (Lindström & Ranta, 1992).

Several studies have indicated that goby nests potentially signal male condition and male parental ability, qualifying them as part of an extended phenotype. Male common gobies build less elaborate nests and lose more eggs when deprived of food (Jackson, Rundle, & Attrill, 2002; Kvarnemo, Svensson, & Forsgren, 1998). In closely related sand gobies (*Pomatoschistus minutus*), nest‐building effort is repeatable (Japoshvili, Lehtonen, Wong, & Lindström, 2012) and increases with male condition (Lehtonen & Wong, 2009; Olsson, Kvarnemo, & Svensson, 2009), although the effect was lost over time in Lehtonen and Wong's study. Nest takeovers are frequent (Lindström, 1992; Lindström & Pampoulie, 2005; Magnhagen, 1992), potentially diminishing the reliability of nests as indicators of male condition (Björk & Kvarnemo, 2012). Nest‐building effort varies with environmental conditions, including the presence of predators (Lehtonen, Lindström, & Wong, 2013) and competitor males (Svensson & Kvarnemo, 2003), the prevailing oxygen concentration (Lissåker & Kvarnemo, 2006), salinity (Lehtonen et al., 2016), and male size (Lehtonen et al., 2013).

Female gobies apparently discriminate among potential partners based on multiple cues such as nest size and male body size (Heubel, 2018; Lehtonen, Rintakoski, & Lindström, 2007). However, the importance of nest modifications beyond the size of the nest per se remains unclear. Correlative evidence suggests that common goby females prefer males that pile up more sand around and on top of their nest (Kalb, Lindström, Sprenger, Anthes, & Heubel, 2016). Only two studies on gobies to date have assessed the role of nest traits independent of male phenotypes by manipulating sand amounts on top of the nest, finding a female preference toward nests with higher sand covers (i.e., larger amount of sand on top) in one (Jones & Reynolds, 1999a) but not in the other (Lehtonen & Wong, 2009). Such inconsistent findings may arise when mate preferences vary with environmental conditions. Under oxygen stress, for example, common goby females have reversed their preference for nests with eggs (Reynolds & Jones, 1999) and lost their preference for well‐built nests, that are covered with a sand pile and have a narrowed nest entrance (Jones & Reynolds, 1999a).

Most previous studies examining the effects of goby nest quality did not differentiate between different components of nest building, nest coverage versus nest entrance width in particular. Narrow nest entrances and high sand cover both provide protection from predators through camouflage and increased defensive structure, but narrow entrance additionally challenges successful egg ventilation. Smaller entrances require more effort for displacement fanning to maintain high oxygen levels (Jones & Reynolds, 1999b), so the nest holders may sometimes have to trade‐off ventilation against nest protection (Lissåker & Kvarnemo, 2006; Olsson, Kvarnemo, Andren, & Larsson, 2016). Indeed, previous studies differentiating nest cover from nest entrance width revealed females to prefer high sand cover but not narrow entrance (Svensson & Kvarnemo, 2005). Moreover, entrance width was affected more strongly than cover height by oxygen levels (Jones & Reynolds, 1999b; Olsson et al., 2016) and water temperature (Olsson et al., 2009). The interests between the sexes might also differ: Females may prefer well‐built nests that better ensure offspring survival and indicate male quality (high sand cover and narrow entrance), whereas males may focus more on making sure that egg ventilation is not too costly.

To our knowledge, no earlier study has disentangled the effects of nest cover and entrance width on female and male nesting preferences using direct experimental manipulation. We therefore experimentally isolated the effects of nest cover height and entrance width using artificial nest models for male nest occupation and female mating decisions. In experiment 1, we tested the preference of male common gobies and its repeatability when selecting among alternative potential nest structures with opposing characteristics. We predicted males to prefer structures that would minimize requirement of additional nest building while ensuring successful nest ventilation, and thus high sand covers and wide nest entrances. In experiment 2, we tested female preferences for nest characteristics independent of nest holder male identity. We predicted that females would prefer high sand cover for its direct benefits in predator protection and possible direct/indirect benefits as a signal of male quality, and narrow entrances as an honest advertisement of male condition as well as protection against predators.

## 2 METHODS

### 2.1 Fish handling and maintenance

We conducted two consecutive experiments to examine male and female preferences for nest traits, respectively, at Tvärminne Zoological Station (University of Helsinki, Finland) during the 2014 breeding season spanning June and July. Common gobies were collected from the shallow bay at Henriksberg Nature reserve (Högholmen, Sandvik) using a seine and hand nets. Nearly, all males used in the experiments were guarding a nest at the time of capture and thus presumably motivated to build a nest. Females were visibly ripe with a round belly (late ripeness, R3 sensu Mück and Heubel (2018)) and therefore ready to spawn. Males and females were housed separately in three 100‐liter stock tanks housing 20 to 40 fish each for at least 24 hr and for not more than a week before being allocated to their experimental treatment. All aquaria were connected to a flow‐through system with brackish seawater and exposed to a natural light regime. During trials, each fish was fed 3–5 frozen chironomid larvae twice per day, and the fish in stock tanks received approximately the same amount of food per individual. Upon entering the trials (males were measured at the start of experiment 1 and females at the start of experiment 2), wet weight of individuals blotted on moist tissue paper (±10 mg, using a precision balance) and total length (±1 mm, using a measuring board with a mm scale) from the tip of the snout to the end of the tail were measured for each fish. To facilitate individual recognition, we injected males dorsolaterally with a VIE tag (Northwest Marine Technology, USA, green/red/pink/blue) after 30s exposure to a 0.04 ‰ clove oil (1:9 eugenol:ethanol)‐sea water solution for anesthesia (Ylitepsa, 2011).

### 2.2 Nest models

**Figure 1:**
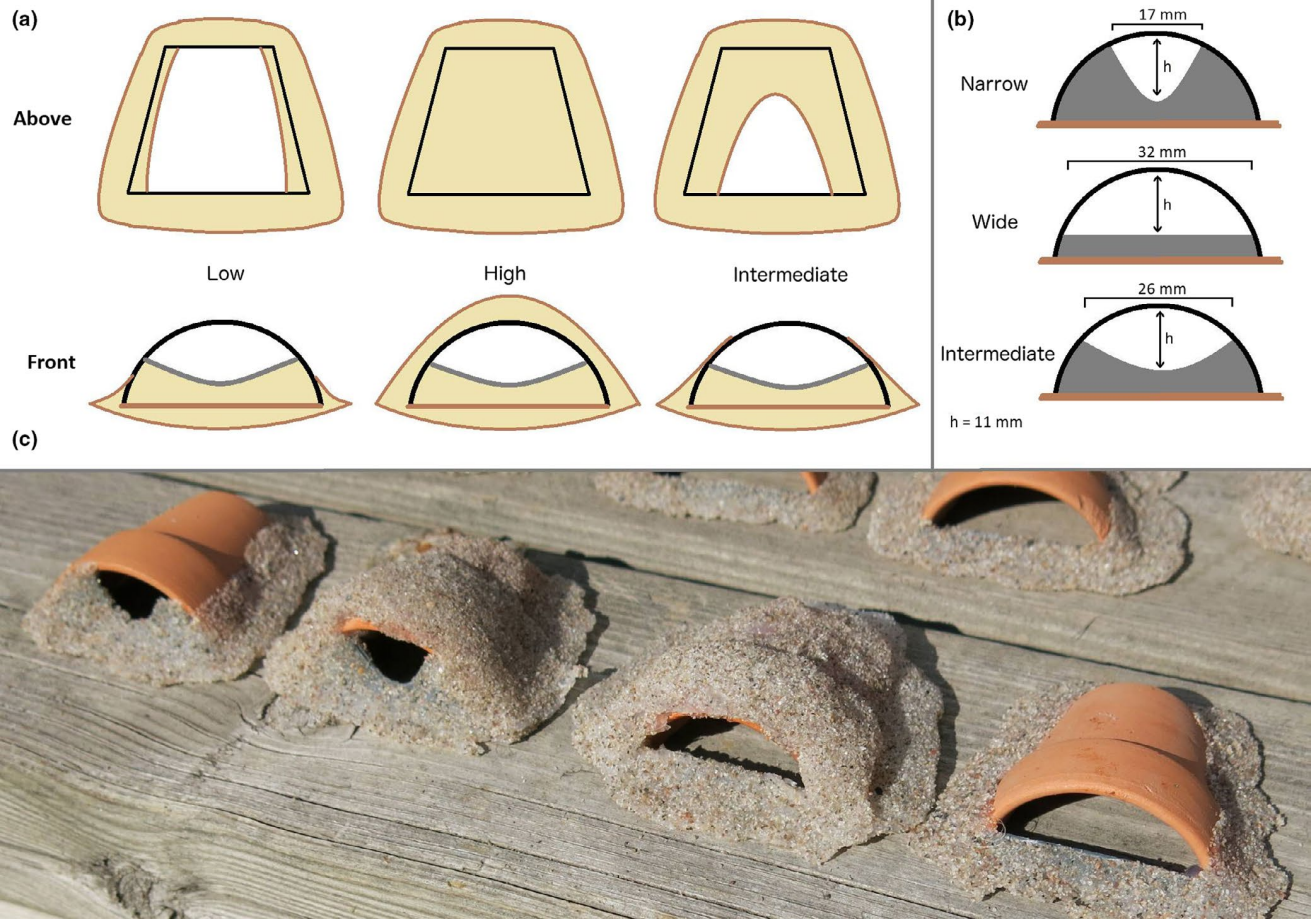
Manipulation schemes for sand cover height (a) and entrance width (b), with exemplary nest models showing narrow–low (NL), narrow–high (NH), wide–high (WH) and wide–low (WL) entrance‐cover combinations used in experiment 1 (from left to right, panel (c))

**Figure 2:**
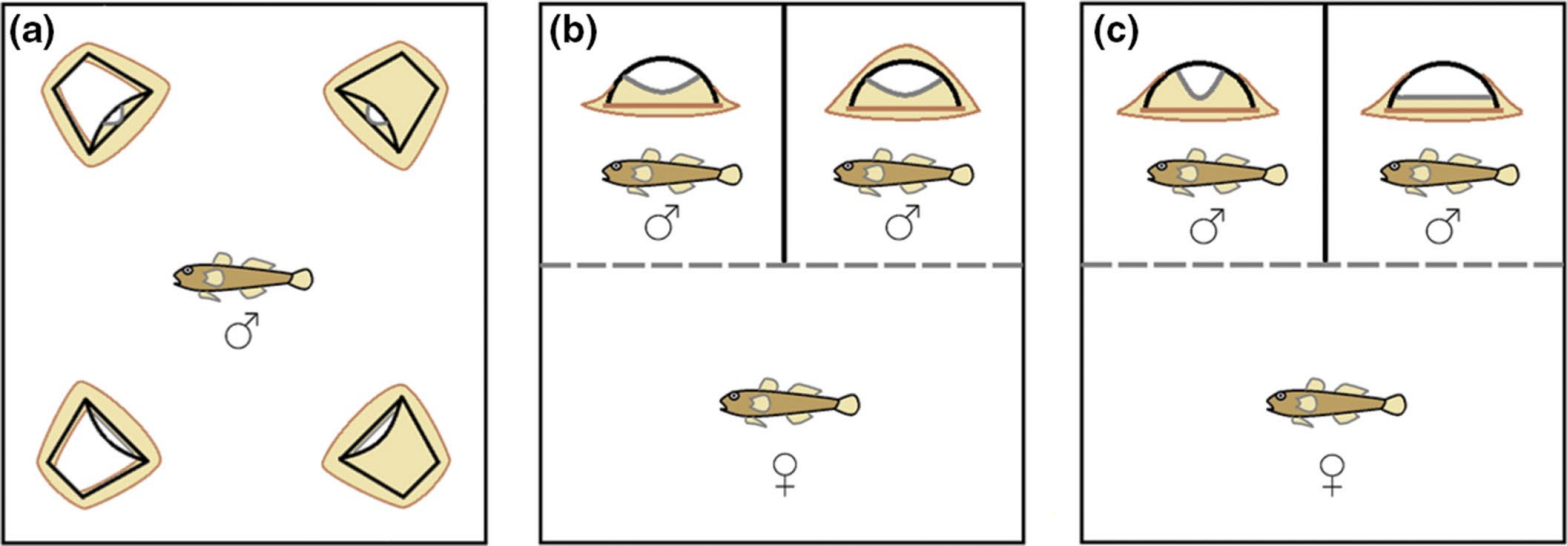
Setup of behavioral experiments for (a) male nest choice (1st trial—only three nests left in 2nd trial with one empty corner), (b) female mate choice with respect to nest cover, and (c) female mate choice with respect to nest entrance width. In b and c, the dotted line represents a transparent divider that was removed after the female had the chance to visually inspect both males

Artificial nests were modeled around halved clay flower pots (38 mm diameter) as the core nesting substrate. We aimed at modeling nest traits within the natural range of nest architecture variation (Japoshvili et al., 2012; Jones & Reynolds, 1999a; Kalb et al., 2016; Kvarnemo et al., 1998; Lehtonen et al., 2016; Lissåker & Kvarnemo, 2006). In contrast to naturally built nests by males where nest appearance usually comes in a coupled set of traits, our use of model nests allowed us to independently modify the two nest traits within the natural range of variation. For *entrance width*, we carved standardized narrow, intermediate, and wide entrances at identical entrance heights from PVC plastic (Figure 1a). For *sand cover height*, we molded a silicon‐sand mix on top of the flower pots as to represent low, intermediate, and high covers (Figure 1b). All nests were finally covered in dry sand atop a silicon base (Figure 1c). In experiment 1, only the four extreme types of artificial nests were used (Figure 2a): narrow–low, narrow–high, wide–high, and wide–low. In experiment 2, females were subjected to a choice either between narrow–intermediate and wide–intermediate, or between intermediate–high and intermediate–low nests (Figure 2b,c).

### 2.3 Experiment 1: Male nest choice

We exposed each of 165 male common gobies to a choice among four types of experimentally manipulated nest substrates. These represented a 2x2 full factorial combination of sand cover height (low, high) and entrance width (narrow, wide), producing narrow–low (NL), narrow–high (NH), wide–low (WL) and wide–high (WH) nest models (Figures 1c, 2a). Each male was tested individually twice, testing the males’ first and second preference for nest architecture variants. The experiment was carried out in 26 black plastic containers (l*w*h = 57*37*30 cm) containing a 2‐cm thick layer of sand to provide males with additional nest‐building material. Each container had its own four nest models, so each nest type had 26 independent replicates of models. Nests were positioned in the four corners of a container in a randomized order.

**Figure 3:**
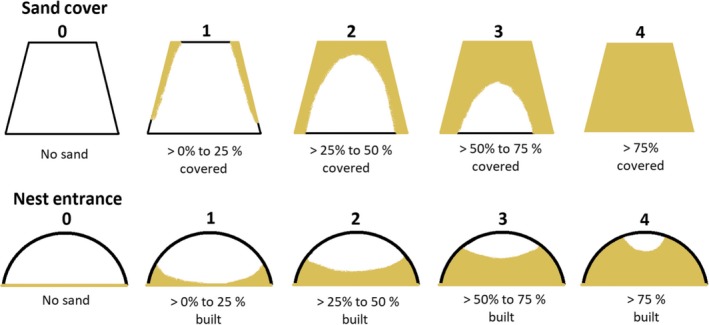
Schematic representation of the scoring scheme for additional nest‐building effort, exemplified using nests with no cover and a wide nest opening (adjusted from Kalb et al., 2016)

Twenty‐four hours after releasing a focal male into the test tank, we scored the additional nest‐building effort dedicated to each of the four potential nests separately for nest entrance and nest cover. These scores estimate the volume of additional sand a male should add to the wide–low nest model to cover a proportion of the entrance or nest roof: 0 = 0%, 1 = 1%–25%, 2 = 26%–50%, 3 = 51%–75%, 4 = 76%–100% (Figure 3). At this time point, nest occupation is usually settled (Heubel, 2018; Magnhagen, 1992) and we further assessed and defined the currently occupied nest as a male's choice in this first experimental trial, with *N* = 142 males exhibiting nest choice.

After this first choice trial, the chosen nest and any sand added to the remaining three nests were removed to check the male's second preference among the remaining nest trait combinations 24 hr later, completed by *N* = 121 males (with a total length of 34.5 ± 2.7 mm and wet weight 420 ± 104 mg [mean ± *SD*]). This second choice round allowed us to also check for male consistency in choices for nest entrance width or cover height, given that the remaining three nest models still comprised all possible traits.

### 2.4 Experiment 2: Female nest and partner choice

Males that had completed their choice tests for their most and second most preferred combination of nest traits as described above entered the female choice trial. Here, we tested either for the effect of nest entrance width (sand height kept intermediate, Figure 2c) or of sand cover (entrance width kept intermediate, Figure 2b). Each replicate contained two males closely matched in size (maximum size difference 2 mm, maximum weight difference 160 mg, mean ± *SD* weight difference 33 ± 36 mg, relative weight difference mean ± *SD* 8 ± 8%, *n* = 61 trials, at an average male weight of 425 ± 97 mg, *n* = 122 males) that were haphazardly assigned to the two treatments, regardless of their preference in experiment 1.

Trials (cover treatment *n* = 31, entrance treatment *n* = 30) were carried out in 12 glass aquaria (six were 40*35*35 cm, six were 50*35*30 cm) with sides covered with black polyethylene foil to prevent visual interaction between tanks. Each tank was separated into a front and a back compartment by a perforated transparent divider. The back compartment was split by an opaque divider to prevent interactions among males (Figure 2b,c). The front compartment had a 2‐cm sand layer to provide females with a burying substrate. In contrast, the back compartments contained only an approx. 1 mm sand layer (approx. 0.5 dl) plus an extra 4 ml within the nests to prevent males from substantial modification of their allocated nests while mimicking natural conditions as closely as possible within the experimental restrictions. Nest ceilings had a transparent plastic sheet attached with a plastic clip to enable egg removal for photographing after spawning. Each tank used its own pair of nest models, so between the two treatments, each nest model type had six independent replicates.

Males were given 24 hr to settle into their assigned nest before placing a female in the front compartment. The transparent divider allowed the female to visually inspect and assess the males and their nests for an hour, before the divider was removed to allow actual spawning. 24 hr later, we checked both nests for eggs. Male nest‐building effort was scored twice, first right before the female was introduced and second when checking for eggs the next day. Any nest‐building effort by the male was removed during the check. If eggs were present in either nest, the transparent plastic sheet with the eggs attached was removed and replaced with an unused one. In the absence of spawning, we immediately initiated a second trial with a ripe replacement female, for a maximum of two times. Following spawning, the female was removed and its body weight remeasured. The female choice trial was then repeated, now with the males swapped between the two nest types and exposed to a novel ripe female. A trial ended when two females had spawned with either of the two males. To minimize observer bias, scoring of male effort and female choice was done blind to the outcome of the previous trial.

For the cover treatment (in brackets: entrance treatment), we initially set up 31 (30) trials with two males each. From these, we excluded instances in which males swapped their allocated nests, potentially confounding our manipulated nest traits with individual male traits (7 cases in the cover treatment and six cases in the entrance treatment). Among the remaining trials, we achieved two consecutive female oviposition events in 16 (20) trials, and a single oviposition event in 10 (4) trials, resulting in a final *N* = 26 trials for the cover treatment, and *N* = 24 for the entrance treatment.

### 2.5 Statistical analyses

#### 2.5.1 Experiment 1: Male nest choice

To assess the effects of manipulated nest entrance and cover on male nest choice, we modeled probabilities of nest occupancy—separately for first and second male choices—using a generalized linear mixed model (GLMM) as implemented in the lme4 package (Bates, Maechler, Bolker, & Walker, 2015) for R version 3.4.2 (R Core Team, 2017). Presence or absence of a given male at each of the four (first choice) or three (second choice) available nests constituted the response variable, modeled with a binomial error distribution. Fixed factors comprised our entrance and cover treatments (two levels each), as well as their interaction. Because nest choice may also vary with effort invested into nest building, we added the sum of nest‐building effort scores for nest entrance and nest cover in a given nest as a *z*‐transformed covariate (total nest‐building effort score) to the model, treated as continuous given that the discrete scores sufficiently approached a normal distribution. Male ID and Tank ID were added as random intercepts to account for the repeated measures per male fish and random variation in nest choice between the experimental tanks, respectively. Data were restricted to males that finished the choice test within the first observation day. Model predictions and their 95% credible intervals (CrI) were derived from posterior distributions of 5,000 model simulations as implemented in the *arm* package (Gelman & Hill, 2007). Model predictions across the range of a given predictor variable were derived for defined values of the remaining model predictors; that is, continuous covariates set to their sample mean when predicting for factorial predictors, and factors set to a specified level when predicting for covariate (Korner‐Nievergelt et al., 2015).

To test within‐individual consistency in male choice for nest traits between first and second trials, we first tested the overall frequency of consistent versus inconsistent choices with respect to either cover height or entrance width against the random expectation of one out of three (33.3%—the “consistent” trait characteristic was still present in one out of the three remaining nest models) using exact binomial tests (*N* = 121 males participating in both choice trials). Second, we tested whether choice consistency varied with the nest cover height and/or nest entrance width chosen during the first choice trial, using GLMM similar to those described above but now with choice consistency (yes or no) for cover height or entrance width as the binomial response.

#### 2.5.2 Experiment 2: Female nest choice

For female nest choice, we modeled choice probabilities separately for the two subsets of the experiment manipulating either nest entrance width or nest cover height as outlined above. GLMMs had the presence or absence of eggs in each of the two available nests as the binomial response variable. The nest entrance (or cover) treatments represented the factorial predictor variable. The models further contained three covariates: (i) total nest‐building effort scores prior to female release (*z*‐transformed), (ii) total nest‐building effort scores at the end of the trial (*z*‐transformed), and (iii) the weight difference between a given nest‐holding male and its competitor. The absence of inappropriate predictor collinearity was assumed when pairwise correlation coefficients did not exceed 0.6. The random component contained the Male IDs (each used twice) nested within Tank IDs (each used for multiple sets of test males). Consistency in choice between the two independent test females for a particular male was assessed using exact binomial tests against the random 1:1 expectation.

## 3 RESULTS

### 3.1 Experiment 1: Male nest choice

**Figure 4:**
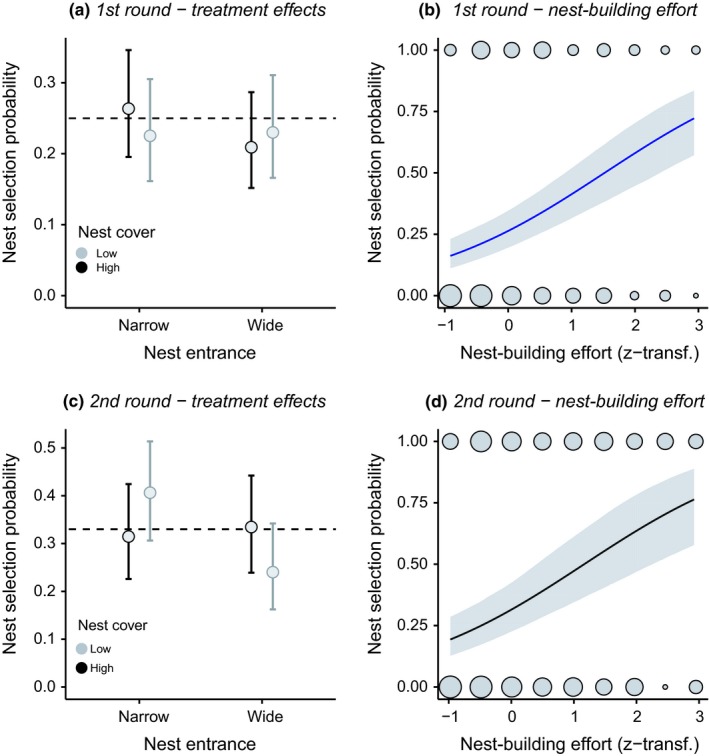
Nest selection in male common gobies. Model predictions ±95% CrI are shown for the first (top row) and second (bottom row) choice trial per male. The left column (a, c) shows how experimental manipulation of nest entrance width and nest cover height affected nest selection when compared to the random choice expectation of 25% and 33% selection probability in the first and second choice run, respectively (hashed lines). The right column (b, d) shows the relationship between nest selection and total additional nest building effort per male. Here, dot sizes indicate raw data frequencies

Across males, we found no indications for an overall preference for either of the two independently manipulated nest characteristics (Figure 4a,c). However, the four model types were well accepted as nests by males. Within trials, at least one of the four nests received a total nest‐building effort score (combined cover and entrance building scores) of ≥2 in 115 of these first choice trials (only one nest received building in 48 cases, two nests in 34, three nests in 18, and all four nests in 15). Nest selection during first as well as second choices was independent of entrance width (GLMM: entrance effect, both *p* > 0.16) and cover height (cover effect, both *p* > 0.84). During second choices, we detected a near‐significant interaction between entrance and cover treatment (χ^2^ = 3.35, *df* = 1, *p* = 0.067), implying that narrow entrances tended to be preferred when combined with low cover, and wide entrances when combined with high cover. In contrast to this mixed pattern, there was a strong positive association between nest choice and the additional sand building effort males invested into a given nest (GLMM: nest‐building effect, both *p* < 0.001, Figure 4b,d).

**Figure 5:**
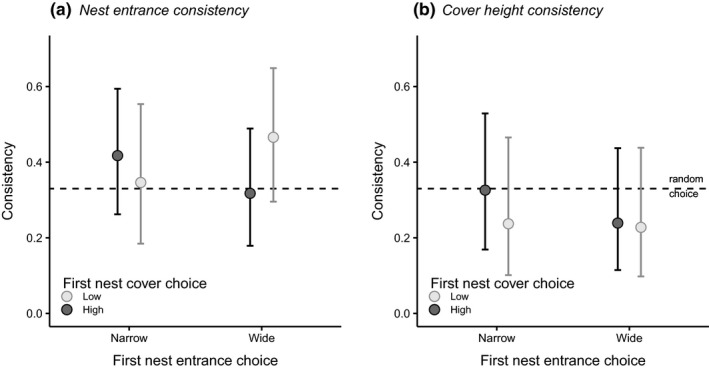
Within‐male consistency in nest selection. Graphs show mean proportions ±95% CrI to which males choose identical nest characteristics in both choice trials, depending on their initial choice and split for nest entrance width (a) and nest cover height (b) (total *N* = 121 males). Dotted lines represent the expected proportion of consistent choices when random (33%)

Within males, we further checked whether individual choices for either nest characteristic were consistent between the two consecutive choice trials. Overall proportions of consistent choices did not depart from the random 1:2 expectation for both nest entrance (exact *p* = 0.611, 95% CrI for choice consistency = 0.301–0.481) and nest height (*p* = 0.702, 95% CrI = 0.218–0.388), and consistency rates were independent of the initially chosen combination of nest characteristics (Figure 5).

### 3.2 Experiment 2: Female nest and partner choice

#### 3.2.1 Female nest preference

**Figure 6:**
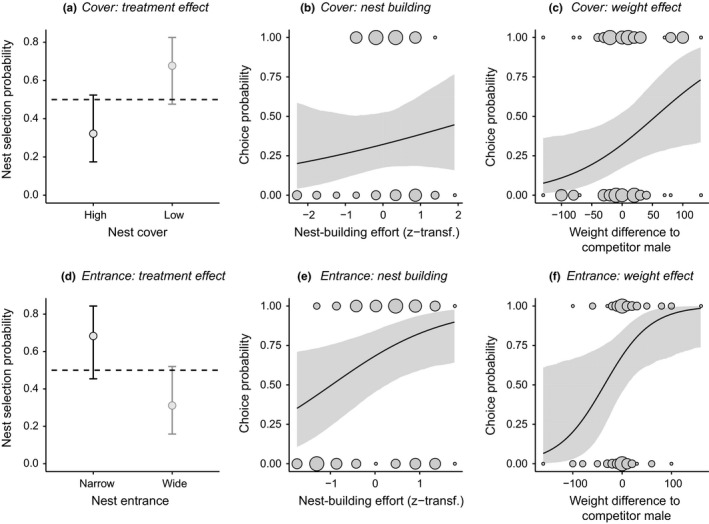
Female choice for nest types and males in female common gobies. Model predictions ±95% CrI are shown for experiments manipulating either nest cover height (top row) or nest entrance width (bottom row). The left column (a, d) shows how the experimental treatment affected female oviposition decisions when compared to the random choice expectation of 50% (dotted lines). Covariate correlations with female choice are shown for total nest‐building effort (central column, b, e) and the weight difference between the two test males (right column, c, f). Here, dot sizes indicate raw data frequencies. Note that positive and negative weight differences are mirrored due to the dyadic nature of the experimental design with each replicate consisting of one chosen and one rejected male

When exposed to a choice between two experimentally manipulated nest types, females significantly preferred low over high nest covers, spawning in low nest models in 26 of 42 cases (Figure 6a, GLMM: cover effect χ^2^ = 5.04, *df* = 1, *p* = 0.025), and narrow over wide entrances, spawning in narrow nest models in 27 of 44 cases (Figure 6d, GLMM: entrance effect χ^2^ = 5.30, *df* = 1, *p* = 0.021). Egg laying probabilities did not covary with the additional sand building effort that males had invested into either nest before females entered the arena (both *p* > 0.35). However, females deposited eggs with higher likelihoods in nests with larger building efforts in the nest entrance experiment (Figure 6b, e, χ^2^ = 4.43, *df* = 1, *p* = 0.035), and with the heavier male in both experiments (Figure 6c, χ^2^ = 3.44, *df* = 1, *p* = 0.064, and Figure 6f, χ^2^ = 4.09, *df* = 1, *p* = 0.043).

Females spawned with the same male in 12 out of 18 cases in the sand height treatment and 18 out of 23 in the entrance treatment, resulting in highly significant choice consistency when pooled across experiments (exact *p* = 0.0043, 95% CrI for choice consistency = 0.571–0.858).

## 4 DISCUSSION

### 4.1 No male preference for specific nest models, but more nest‐building investment at chosen nests

We found no evidence that males discriminated among the offered nest models and thus found no support for the hypothesis that cover height or entrance width constitute relevant components of male nest choice. We can think of five possible explanations. First, nest‐building costs may be negligible and thus induce no need to save resources. Previous studies indeed did not detect costs, such as reduced lipid mass, associated with increased nest‐building activity in gobies (Olsson et al., 2009). In our study, males invested considerably in nest building despite the absence of a female, often simultaneously in multiple nests. Hence, even if costs were present, our mild experimental conditions with regular feeding and the absence of predators may have failed to make them apparent. Second, the modified nest attributes from well within the natural range of nest‐building variation might provide males with too small expected benefits. This could arise if nest attributes had no effect on female attraction, a scenario we can exclude given our findings in the female choice trial. Alternatively, since the fine sand in the natural shallow goby habitat is exposed to permanent water turbulence, the benefits from initial nest attributes may be small in comparison with the constant need of nest maintenance. Third, the above‐mentioned costs and benefits of nest choice may arise only under more challenging environmental conditions, with increased competition or predation risk being plausible candidates in gobies. Similar to many birds (Eggers, Griesser, Nystrand, & Ekman, 2005; Peluc et al., 2008), goby males may prefer more protective nest only when the perceived predation risk is high. Fourth, our nest models may not have captured a scenario of trait combinations that males realistically find in nature. While principally true, our finding that males and females instantly accept nest models as a breeding substrate even without the provision of much additional sand, and the presence of female choice for nest model type suggests a sufficiently realistic setting. On a similar note, our timing of assessing male nest choice decisions only once after 24 hr may have been not sufficient. Finally, male nest choice may rest on other traits than those manipulated in our study. Previous studies with similar setups found goby males to prefer larger over smaller nesting substrates (Flink & Svensson, 2015; Japoshvili et al., 2012; Lindström, 1988), so size may contribute more to male preference than entrance width or cover height. While we cannot differentiate between these alternative explanations for the absence of male nest choice, our data confirm that nest building per se appears important: All males substantially invested in nest building and typically resided in the nest that had received most building effort.

### 4.2 Females preferred specific nest types, increased male effort, and heavier males

As predicted, females preferred nests with narrow entrances. To our best knowledge, no earlier study has reported female preferences for nest opening width when separated from alternative nest or individual male traits. For gobies, this preference has possibly gone undetected because the entrance can usually be easily modified by the male and may thus change throughout the experiment (Svensson & Kvarnemo, 2005). While the solid model nest entrances used in the present study might not perfectly represent natural conditions, the detected female preference can still bear ecological implications. For example, preference for constantly narrow nest openings might raise the risk of inadequate levels of oxygen being provided to the developing eggs. Goby males have previously prioritized successful nest ventilation regardless of the presence of predators (Jones & Reynolds, 1999b; Lissåker & Kvarnemo, 2006; Olsson et al., 2016), suggesting a possible conflict of interests with nest protection being a priority for females but easy maintenance for males. Males might therefore build a narrow entrance to impress females, but widen the nest entrance after the female has spawned. Indeed, nest entrance width has previously been recorded as constantly changing (Svensson & Kvarnemo, 2005) and less repeatable than cover building (Japoshvili et al., 2012) in sand goby males. Stickleback females prefer compact nests only at high oxygen levels while preferring males in good condition irrespective of oxygen level (Head et al., 2017) and common goby females might behave similarly.

Contrary to our prediction and findings of previous studies (Jones & Reynolds, 1999a; Svensson & Kvarnemo, 2005), females preferred low rather than high sand cover. This discrepancy could be reconciled when it is not the absolute amount of sand cover, but the degree of nest camouflage under the current environmental conditions that determines female choice as suggested for several bird species (Kleindorfer, 2007; Stevens, Troscianko, Wilson‐Aggarwal, & Spottiswoode, 2017). Our experiment was special in placing artificial nests onto an aquarium bottom almost devoid of extra sand to prevent further nest building. In such an environment, nests with high sand cover may have stood out in an exaggerated manner. If nests acted as a male ornament, as for example in bower birds (Borgia, 1985), exaggerated nest attributes like this might have reinforced female choice, but we found no support of this in our study. Overly concealed nests may also limit visibility for predator detection, as has been suggested in a study where White‐rumped shamas selected and had a higher nesting success in less‐concealed nest boxes (Chotprasertkoon et al., 2017).

Our experimental manipulation reveals that nest characteristics affect—at least to some degree—female choice beyond the nest holder phenotype, similar to results by Jones and Reynolds (1999a) (but see Lehtonen & Wong, 2009). Contrary to earlier studies, our experimental setup left only minimal room for male behavior to confound any treatment effects. While pretrial male nest‐building effort did not affect female choice, we found the chosen nests to have higher nest‐building effort scores when the mate choice trials were terminated. We cannot distinguish whether this extra nest‐building effort occurred prior to female spawning, then leaving the option that females were also attracted to building behavior as a potentially honest male quality signal (Zahavi, 1975), or after spawning, when nest amendments were linked to another trait like displacement fanning (Olsson et al., 2009; Svensson & Kvarnemo, 2005). Preferred males would thus invest more in nest building as part of their brood care similar to observations in birds and spiders (DiRienzo & Aonuma, 2018; Soler, Cuervo, Møller, & DeLope, 1998).

Despite this modest effect of nest attributes beyond male characteristics, we found that different females consistently preferred the same male individual in consecutive choice trials, regardless of the nest model type it resided in. Male identity thus affected female mating decisions more strongly than nest attributes. However, we cannot fully rule out that a male's initial mating success (followed by the experimenter's egg removal) changed a male's behavior in a way to increase his chances to secure a second mating with another naïve female. Consistency in female mate choice is by no means universal (Jennions & Petrie, 1997). Previous research found weak consistency in female sand gobies (Lehtonen & Lindström, 2008), but rather strong consistency linked to male body size in the same common goby population as studied here (Kalb et al., 2016). Our current study again reveals a strong correlation between male body weight and female preference. Large males are known to be preferred in particular under perceived risks of female–female competition (Heubel, 2018) or a chance of male–male competition (Lehtonen & Lindström, 2009), with the latter scenario nicely matching our experimental choice setup.

Combined, our findings provide direct evidence for a mild contribution of nest characteristics to female mate choice and correlational support for a major role of the nest holder male's phenotype, specifically body size and possibly sand building effort. Hence, female preference in common gobies likely relies on multiple and perhaps complementary cues. Mate choice based on multiple cues has been suggested to evolve in changing environments where the reliability and expression of solitary signals may change easily (Bro‐Jørgensen, 2010). This applies well to the common goby, since nest material abundance (and thus the intensity of intrasexual competition (Forsgren et al., 1996)) as well as sex ratio (Mück & Heubel, 2018) can vary locally, and water temperature can change dramatically within a single breeding season (personal observation, 2014). Since female and male reproductive behavior of gobies also changes over the mating season based on temperature (Kvarnemo, 1996), nest availability (Borg, Forsgren, & Magnhagen, 2002), and mate availability (Heubel, Lindström, & Kokko, 2008), some cues might also play a larger role depending on the environmental conditions or social context (de Jong et al., 2018). In the European bitterling, females approach males after assessing their phenotype, but take their final mating decision only after inspecting the nest (Candolin & Reynolds, 2001). Goby females may perform a similarly sequential decision, but whether they use the nest or the male phenotype as the first cue might depend on environmental context: Under more intense female–female competition for example, females might have to make quick decisions based on nest attributes instead of male phenotype (Heubel, 2018).

## 5 CONCLUSIONS

In this study, we experimentally manipulated goby nest attributes to investigate male nesting and female mating preferences. While male nest choice was unaffected by variation in nest entrance width and cover height, we show that females prefer narrow over wide entrances, probably for their added protection against predation. Given that—under natural conditions—these characteristics originate from male nest building and maintenance activities, nest attributes may qualify as an extended male phenotype. Yet, our study also highlights the relevance of male body size, suggesting that female mate choice rests on multiple cues. Nest models turned out as a powerful tool to disentangle these preferences from confounds of individual phenotypes and thus appear promising for further studies exploring the added effects of variation in food availability, dissolved oxygen, salinity, or predation pressure on nest choice.

## CONFLICT OF INTEREST

The authors declare that they have no competing interests.

## AUTHOR CONTRIBUTIONS

VP, KH, NA, and MV designed the experiment. VP, NK, KH, and MV conducted field work. VP and NK collected the data. VP, NA, and KH contributed in data analyses. VP and NK drew illustrations on setup. VP wrote the first draft of the manuscript. VP, NA, and KH contributed in drafting of the manuscript. All authors have substantially contributed to the work and revised the manuscript and read and approved the final version.

## ETHICAL APPROVAL

The study complies with all the relevant laws of Finland and was approved by Finnish authorities. All procedures were declared as class 0 experiments and inspected and approved by ELLA, Animal Experimental Board in Finland on site at Tvärminne zoological station in Hanko, Finland.

## Data Availability

The datasets generated during and/or analyzed during the current study are available from the Dryad Digital Repository: http://dx.doi.org/10.5061/dryad.27nj856.
